# Evaluation of the Shear Bond Strength of Immediate and Delayed Restorations of Various Calcium Silicate-Based Materials with Fiber-Reinforced Composite Resin Materials

**DOI:** 10.3390/polym15193971

**Published:** 2023-10-02

**Authors:** Merve Candan, Fatıma Kübra Altinay Karaca, Fatih Öznurhan

**Affiliations:** 1 Department of Pedodontics, Faculty of Dentistry, Eskişehir Osmangazi University, Eskişehir 26040, Turkey; 2 Department of Pedodontics, Faculty of Dentistry, Sivas Cumhuriyet University, Sivas 58140, Turkey

**Keywords:** calcium silicate cements, fiber-reinforced composites, shear bond strength, dental materials, TheraCal PT, NeoPutty, NeoMTA2, pediatric dentistry

## Abstract

Due to significant tissue loss in teeth requiring pulp treatments, hermetic restoration of the remaining dental tissues is one of the most crucial factors in determining the treatment’s success. The adhesion of composite resins to calcium silicate cements (CSCs) is considered challenging. Consequently, it is crucial to identify the optimal method for obtaining optimal adhesion. The aim of the present study is to evaluate the shear bond strength (SBS) values of immediate and delayed restorations with fiber-reinforced composites on powder–liquid, premixed, and resin-containing flowable CSCs. In the present study, the SBS values obtained after immediate (14 min) and delayed (7 days) restorations of three different CSCs (NeoMTA2, NeoPutty, and TheraCal PT) with three different resin composite materials (EverX Flow^TM^, EverX Posterior^TM^, and Filtek Z550) were compared. The fracture types were evaluated using a stereomicroscope and SEM. TheraCal PT had the highest SBS values for both immediate and delayed restorations, and the comparison with other materials showed a statistically significant difference (*p* = 0.001). In contrast, there was no statistically significant difference between the SBS values of NeoMTA and NeoPutty (*p* > 0.05). In both immediate and delayed restorations, there was no statistically significant difference between nanohybrid and fiber-reinforced composites (*p* > 0.05). The simple use and strong bonding ability of TheraCal PT with composite resins may provide support for the idea that it is suitable for pulpal interventions. Nevertheless, due to the in vitro nature of this study, additional in vitro and clinical studies are required to investigate the material’s physical, mechanical, and biological properties for use in clinical applications.

## 1. Introduction

Increasing awareness of minimally invasive treatments is crucial not only for hard tissues but also for the preservation of pulp tissue and long-term tooth retention. To maintain the integrity of pulp tissue, it is imperative to carefully evaluate the treatment modalities, select appropriate dental restorative materials (DRMs), and assess the restorability of the remaining dental tissues. Since high-biocompatible calcium-silicate-based materials (CSBMs) were introduced, they are being used more for pulpal therapies [1].

Vital pulp therapy is a conservative treatment modality that seeks to preserve the vitality and functionality of the dental pulp that has been exposed due to caries or trauma. The management of pulp exposures in immature permanent teeth is regarded as an effective treatment modality because it eliminates the need for more invasive therapeutic interventions [2]. Mineral trioxide aggregate (MTA) is a CSBM that is extensively employed in many endodontic operations, including pulp regeneration, hard tissue repair, perforation repair, and root-end filling [3]. This bioactive substance exhibits notable effects on surrounding tissues [4,5]. Despite the notable laboratory and clinical efficacy, including the biological sealing, biocompatibility, and stimulation of hard tissue deposition demonstrated by MTA [6], there are some limitations that impede its clinical application. These limitations include the discoloration of dental tissues after treatment, the difficulty of manipulation, the need for specialized instruments during application, and the slow setting time [4,7]. Consequently, efforts are being made to develop and continue developing cements that incorporate different forms of calcium silicates to overcome these limitations. In addition to powder–liquid-type CSBMs, which are widely used today, premixed and flowable-type CBSMs that can be applied directly to the cavity have been developed to get around these problems [8,9,10].

The majority of teeth requiring pulp therapy exhibit substantial tissue loss. Therefore, the preservation of the pulp by covering it with a biocompatible material and the hermetic restoration of the remaining dental tissues are the most important factors in determining the success of the treatment. Microleakage induced by the chipping/breaking of composites and polymerization shrinkage in teeth with extensive coronal damage negatively impact the treatment’s prognosis [11].

Today, short-fiber-reinforced composites (SFRCs), which can be used as base and restoration materials in dentistry applications, offer advanced physical properties compared to particle-filled resin-based dental composites [12]. The preference of SFRCs to support the remaining dentin tissue and restore lost dentin tissue is one of the biomimetic approaches that can be utilized for the restoration of severely damaged teeth [13,14,15,16]. Resistance to chewing forces is an essential factor for the success of posterior dental restorations. According to the literature, the use of SFRCs as the base material can prevent fractures in the restoration due to the efficacy of the composite fibers in preventing cracks [13].

The adhesion of composites to CSBMs used for vital pulp treatments is considered to be challenging. Therefore, it is essential to identify the optimal composite–CSBM combinations for optimal adhesion [17,18]. Advancements in technology have facilitated the incorporation of various nanofillers, resin matrices, and fibers into DRMs. Additionally, novel adhesion protocols, application methods, and restoration techniques are being devised. Therefore, it is necessary to investigate the mechanical/physical properties of newly developed fiber-reinforced composite polymers, such as particle-containing composites, for their clinical application with other restorative materials.

Various forms and contents of CSBMs have been produced for many purposes, such as shortening treatment times and facilitating their application in pediatric dentistry [3,7]. In this context, many studies have been conducted on CSBM adhesion to teeth and restorative dental materials and their mechanical properties. However, a review of the literature revealed that there were very few studies on immediate and delayed restorations of CSBMs with distinct contents [19,20,21,22]. To the knowledge of the authors, this is the first study to evaluate the bonding strength achieved through immediate and delayed restoration with fiber-reinforced composites to the various CSBMs used in the present study. Consequently, the aim of the present study is to evaluate the shear bond strength (SBS) values between powder–liquid, premixed, and resin-containing flowable CSBMs frequently used in vital endodontic treatments and nanohybrid and fiber-reinforced composites, which are applied to improve the structural stability of teeth damaged by extensive caries.

## 2. Materials and Methods

The researchers acquired the necessary ethical approval for the current study from the Non-Interventional Clinical Research Ethics Committee of Sivas Cumhuriyet University (Approval number: 2023-04/46).

### 2.1. Study Design

In the present study, 18 subgroups were formed based on the restorations of three different resin restorative materials to three different CSBMs performed in two different time periods (immediate-delayed restoration). When α = 0.05, β = 0.10, and (1−β) = 0.90, it was decided to include 180 samples (*n* = 10). The test’s power was determined to be *p* = 0.90729. Table 1 details the type, composition, and manufacturer of the DRMs and CSBMs used in this study.

As a template for the acrylic molds required for inserting samples in the universal testing machine (LF Plus, LLOYD Instruments, Ametek Inc., Fareham, UK), a cylindrical metal mold with a 1.25 cm diameter and a 1.30 cm height was fabricated. In the center of the upper surface of the fabricated metal mold, a cylindrical cavity (depth: 2 mm, diameter: 4 mm) was formed for the placement of CSBMs. Consequently, acrylic molds were made using this metal mold. After separating the prepared acrylic molds into three subgroups, the CSBMs were placed in the molds and polymerized per their manufacturer’s instructions. To mimic immediate and delayed restorations, samples from each group of prepared CSBM were divided into two equal groups.

The powder and liquid components of NeoMTA 2 were mixed as per the manufacturer’s recommended powder–liquid ratios. The premixed dough-like Neoputty and the prepared NeoMTA2 were placed in molds and polymerized at 37 °C and 100% humidity, per the manufacturer’s recommendations. The TheraCal PT (ThclPT) samples were polymerized in accordance with the manufacturer’s instructions using a light-emitting diode (LED) device (Elipar S10 LED Light Device, 3M ESPE, St. Paul, MN, USA) with 1200 mW/cm^2^ light power and 430–480 nm wavelength light. In a similar manner, the polymerized ThclPT materials were subjected to storage conditions of 37 °C and 100% humidity until immediate or delayed restoration, using the same protocol as other materials.

NeoMTA 2 has a similar chemical composition to NeoPutty, and according to the manufacturer’s instructions, the polymerization time ranges from 14 to 70 min. In light of this circumstance, the present study determined time periods of 15 min for immediate restoration and 7 days for delayed restoration. The 7-day time period was determined based on previous studies in the literature [21,22,23].

Following the removal of the CSBMs from the incubator, surface polishing was not conducted. After all sample surfaces were washed with water for 5 s using the air–water spray of the dental unit, the moist sample surface was dried for 5 s. Universal adhesive (3M Espe Scotchbond Universal Plus Bonding) material was applied by rubbing the entire surface of the sample with a disposable bonding brush. The adhesive was then air-dried for at least 5 s until there were no visible fluctuations. The bonding agent was polymerized for 20 s using a 3M Elipar S10 LED Light Device (3M-ESPE, St. Paul, MN, USA), producing light with a power of 1200 mW/cm^2^. After the polymerization of the bonding agent, a transparent polyethylene tube (diameter and height of 2 mm) was set in the center of the CSBMs in each acrylic block. Then, each DRM was placed in a tube with a thickness of 2 mm and light-cured in accordance with the manufacturer’s recommendations. After the polymerization process of the materials was complete, the transparent tube was carefully cut in the vertical direction with a scalpel and removed. Using the universal testing machine (LF Plus, LLOYD Instruments, Ametek Inc., Fareham, UK), the SBS values of the samples were determined.

### 2.2. Shear Bond Strength Testing

A universal testing machine (Lloyd LF Plus, Ametek Inc., Fareham, UK) was used for SBS testing in the present study. The specimens were fixed in the custom frame of the machine, and a constant crosshead speed of 1 mm/min was applied to the adhesive interface of the materials until failure occurred. The test was automatically terminated upon the occurrence of a fracture, and the results were calculated in megapascals (MPa).

### 2.3. Evaluation of Stereomicroscope Images of the Interface between the Calcium Silicate and Composite Materials after the SBS Test

After the SBS test, the fractured surfaces of all samples were examined under a stereomicroscope (SMZ 800, Nikon, Tokyo, Japan) at 25× magnification. The fracture types of the samples were determined and recorded following the examination. The fracture pattern was classified in the following manner: adhesive-fracture (failure between the CSBM and the composite, with no remnants of resin), cohesive-fracture within the material, and mixed-fracture (comprising both cohesive and adhesive fractures).

### 2.4. Scanning Electron Microscopy (SEM) Analysis

SEM analysis was performed on representative samples of the most frequently observed fracture type in each CSBM group. The samples were adhered to the aluminum block using adhesive tape, placed in the Quorum Q150RS Plus Sputter Coater (today), and coated with 5 nm of gold. The same calibrated operator scanned the entire surface and evaluated samples at 10 kV at 40×, 100×, 500×, and 1000× magnification. 

### 2.5. Statistical Analysis

The statistical analysis of the data obtained from the present study was carried out using SPSS 22.0 (SPSS Inc., Chicago, IL, USA). When the parametric test assumptions were not fulfilled (the Shapiro–Wilk test), in the evaluation of the data, the Kruskal–Wallis test was used when comparing the measurements obtained from more than two independent groups. Since the assumptions of the parametric test had been fulfilled during the data evaluation, variance analysis was utilized to compare the measurements obtained from more than two independent groups. Tukey’s test was used to determine which groups differed as a result of the analysis, whereas the paired sample *t* test was utilized to compare two measurement values derived from the same materials. *p*-values equal to or less than 0.05 were considered statistically significant.

## 3. Results

### 3.1. The General Comparison of Composite Groups for Immediate and Delayed Restorations

In general, when the SBS values of immediate restorations made with composite resin materials were compared, there was no statistically significant difference between composite materials (*p* = 0.740). Similarly, there was no statistically significant difference between composite materials when the SBS values of delayed restorations made with composite resin materials were compared (*p* = 0.508). 

### 3.2. The Comparison of Calcium-Silicate-Based Material Groups for Immediate and Delayed Restorations

In the present study, a statistically significant difference was observed when comparing the SBS values obtained from immediate restorations of CSBMs (*p* = 0.001*). There were statistically significant differences between the mean SBS values of the ThclPT group (34.97 ± 7.30 MPa) and the other CSBM groups (*p* ≤ 0.05). However, no statistically significant difference was found between the mean SBS values of the NeoPutty (4.53 ± 1.25 MPa) and NeoMTA2 (4.16 ± 1.42 MPa) groups (*p* > 0.05).

Similarly, when the SBS values determined after delayed restorations of CSBM groups were compared, a significant difference was found (*p* = 0.001*). There were statistically significant differences between the mean SBS values of the ThclPT group (25.95 ± 7.70 MPa) and the other CSBMs (*p* ≤ 0.05). However, no statistically significant difference was found between the mean SBS values of the NeoPutty (12.27 ± 4.61 MPa) and NeoMTA2 (10.81 ± 5.36 MPa) groups (*p* > 0.05). 

### 3.3. The Evaluation of Failure Types

**Figure 1:**
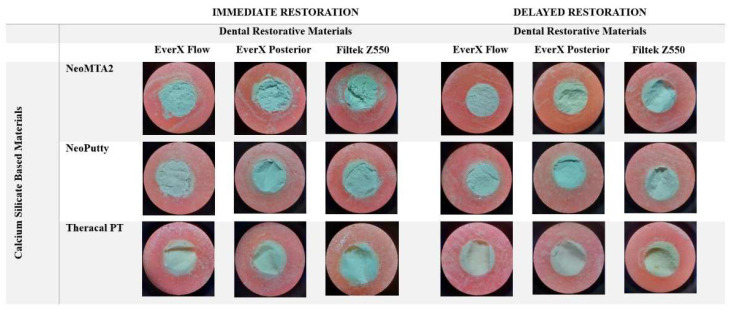
Representative stereomicroscope images of the most prevalent fracture type in each adhered material group.

**Figure 2:**
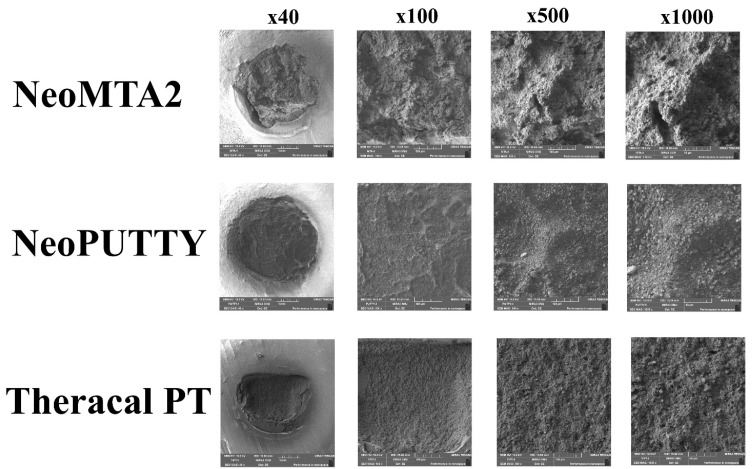
Representative SEM images of the most prevalent fracture type in each CSBM group.

Table 2 displays the SBS values obtained for immediate and delayed restorations of all tested material subgroups. The evaluation of failure types that occurred following the SBS test was conducted in this study, and the associated results are presented in Table 3. Cohesive failure was established as the prevailing failure type across the various examined material groups. Figure 1 and Figure 2 display representative stereomicroscope photographs and SEM images, respectively, illustrating the prevailing fracture type within each group.

## 4. Discussion

The construction of fiber-reinforced composites plays a key role in adhesion-based unibody dental restorations [14]. The physical properties of short-fiber-reinforced composites, particularly fracture resistance and flexural strength, are higher than those of particulate-filled resin-based composites [12]. In addition, the failure mode and load-bearing capacity of restorations made with SFRCs as the base material were reported to be superior to restorations made with conventional composite alone. Therefore, SFRCs can be recommended as an alternative to indirect restorations for large cavities [13,14]. 

MTA was developed and proposed because extant root-end filling materials lack “ideal” properties. Nevertheless, this material is accompanied by some disadvantages, including the possibility of tooth discoloration, a longer setting time, reduced compressive strength, and challenging handling [6,22]. NeoMTA2 is a CSBM that has been created to address the aforementioned drawbacks. It is a nontoxic bioactive substance that exhibits superior handling properties, is resistant to washing away, and does not discolor the tooth [9]. It has been reported that premixed CSBMs do not discolor teeth, are ready for use without the need for additional mixing, and exhibit similar physical/chemical properties to MTA with improved handling properties. NeoPUTTY is a commercially available bioceramic material that has been premixed and contains bioactive properties. It is composed of tri- and dicalcium silicate particles, which are the same components found in NeoMTA2. It has also been reported that the firmness, non-stickiness, resistance to washing, bioactivity, and ability to use with no waste make this material more desirable in the clinical setting [10].

Light-cured resin-modified CSBMs are a promising material for pulp capping because they can be placed exactly where they need to be, have better physical strength, are less soluble, and release fewer heavy metals [24]. The recently developed ThclPT is a biocompatible, resin-containing calcium silicate material. Its chemical composition consists of calcium silicate particles in a hydrophilic matrix that facilitates Ca^+2^ ion release. It has been stated that this pulpotomy and pulp capping material protect the tooth’s vitality by functioning as a barrier and protection for the dental pulp complex [25]. Resin-containing CSBMs have greater cytotoxic effects than non-resin-containing cements, which raises some concerns regarding their use in vital pulp treatments [24]. However, TheraCal LC (ThclLC), the predecessor of ThclPT, was reported to be comparable to Biodentine and MTA HP in terms of the grade of dentin formation in the dentin bridge in a previous study [5]. According to recent studies, ThclPT has higher in vitro mineralization potential and cytocompatibility in human dental pulp stem cells (hDPSCs) compared to ThclLC. It was also reported that it offers comparable biological properties to other hydraulic CSBMs, such as MTA, NeoMTA, and Biodentine [26,27]. ThclLC, a light-curing CSBM, has been reported to have limited clinical performance as a direct pulp capping agent, particularly when evaluated over the long term, according to a meta-analysis study published in 2022 [28]. The newly developed ThclPT and ThclLC share some clinical indications for use, but their manufacturers recommend using ThclPT for pulpotomies and ThclLC as a liner or direct/indirect pulp capping agent, respectively [25].

Various tests, such as SBS, microshear bond strength (mSBS), and microtensile bond strength (mTBS), are used to evaluate the bonding strength of DRMs with teeth or other materials [21,22,29,30]. Compared to the conventional SBS test, the mTBS and mSBS tests permit the selection of standard tooth regions. The material to be applied to the base material prepared for the mSBS test is applied to the polyethylene tube molds with a smaller diameter than those used in the SBS test. Therefore, caution must be taken to avoid cracks and fractures when removing the polyethylene tube after sample preparation. Contrary to the SBS and mSBS tests, to perform the mTBS test, it is necessary to take sections of specific diameters from the prepared samples. There are numerous techniques for sectioning, but unexpected microcracking can occur during sectioning. As a result, the SBS test was favored in the present study due to its simplified test protocol and direct sample preparation [30,31].

Resin composites can be adhered to CSBMs used in vital pulp treatments using etch-rinse or self-etch adhesive systems. Nevertheless, according to a previous study, acid-etch procedures have a negative effect on the microhardness and surface microhardness of ProRoot MTA during the early stages of polymerization. For this reason, scholars have proposed that a minimum waiting period of 96 h should be observed after the mixing of MTA prior to the utilization of acid-etch adhesive systems [32]. To assure standardization, a universal adhesive used in self-etch mode was selected over an acid-etching adhesive system in the present study.

There are numerous mechanisms for the polymerization of CSBMs. CSBMs of a powder–liquid or already mixed form can self-cure, while CSBMs that contain resin can be either dual-cured or light-cured. Consequently, numerous researchers have focused on determining the optimal duration necessary for the restoration of self-curing CSBMs [19,20,21,22,33]. While several investigations in the literature have indicated that there is no significant difference in the SBS of composites to CSBMs with varying polymerization periods [19,33], it has been noted that certain CSBMs exhibit higher SBS values with delayed restorations [20,21,22]. Another study suggests that it is more advantageous to perform MTA restoration with a delayed time period [21]. In the present study, it was shown that the delayed restoration of the NeoMTA2 and NeoPutty groups had higher SBS values compared to their respective immediate restoration groups.

Since the CSBMs evaluated in our study are relatively newly manufactured materials, there are few published studies on SBS. According to a study evaluating the mSBS of tricalcium silicate cements with different restorative materials, CSBMs incorporating resin exhibited the highest SBS values for all the restorative materials investigated [34]. In a prior investigation, it was observed that ThclLC presented higher SBS values than MTA. It has been reported that the observed result could potentially be attributed to the resin content present in ThclLC, which has the ability to form a chemical bond with the composite material [35].

Similar to the present study, Falakolu et al. observed that ThclPT had higher SBS values than NeoMTA2 [36]. Ozata et al. noted that TheraCal LC, the successor to Theracal PT, had higher SBS values than NeoPUTTY and NeoMTA2, and they reported that this may be due to the chemical bond that ThclLC formed with the composite resin as a result of copolymerization [37]. Due to the higher SBS values observed in this study, we believe that this may also be valid for ThclPT.

In the present study, significant differences were observed in SBS values between immediate and delayed restorations of the ThclPT material. The higher SBS values in immediate restoration may be attributable to the presence of an oxygen inhibition layer formed after the material has been light-cured [24]. The degradation of this layer over time after light curing of the material may be the reason for the lower SBS values of delayed restorations.

According to Ozata et al., there is no difference between the SBS values of NeoMTA2 and NeoPutty materials [37]. Similarly, in the current study, there was no statistically significant difference between the SBS values of NeoPutty and NeoMTA2 for both immediate and delayed restorations. Nonetheless, it was determined that the average SBS values obtained in the studies varied. This could be due to the variability in the used restorative materials, application and restoration times, storage time, storage media, operators, and adhesive methods [38]. Additionally, SBS values can vary based on the use of various bonding systems and surface treatments [37].

According to a study, variations in fiber orientation have an impact on the propagation of cracks in fiber-reinforced composite resins. However, the same study concluded that there was no statistically significant difference between the SBS values of fiber-reinforced composites and particle-filled composites [15]. In this particular context, it is crucial to evaluate each CSBM in various forms individually, with the aim of determining the most appropriate composites and combinations of CSBMs. In the course of the present study, it was determined that no singular composite material group exhibits significantly higher SBS values with all CSBM groups.

In the majority of studies evaluating the SBS values of materials, the values were compared, but their clinical acceptability was not discussed. Some studies accept 9 MPa [39,40] or 10–13 MPa [41,42,43] as an acceptable SBS value, while others have suggested that this value should be at least 17.20 or 18 MPa [38,44]. In addition, a meta-analysis revealed that the clinically acceptable bond strength value for inter-material (composite–composite) adhesion remains unclear [18]. In conclusion, there appears to be disagreement regarding the clinically acceptable SBS value in the literature. For this reason, only the comparison of SBS values derived from various material groups was conducted in the present study.

To achieve optimal clinical restoration, it is important to establish ideal adhesion between the DRMs. In general, the bond is acceptable when fracture occurs within each DRM rather than at the bonded interface (i.e., cohesive instead of adhesive) [35]. Similarly, cohesive-type fractures were frequently detected among the groups evaluated in the present study.

## 5. Conclusions

Consequently, the results of the present study indicated that there was no statistically significant difference between fiber-reinforced and nanohybrid composite materials SBS values for immediate and delayed restorations of calcium-silicate-based materials. The resin-containing TheraCal PT material exhibited the highest SBS values when compared to the NeoMTA2 and NeoPutty materials. Moreover, higher SBS values can be achieved through immediate restoration of Theracal PT material and delayed restoration of NeoPutty and NeoMTA2 materials. Given this circumstance, the simple application and strong bonding ability of TheraCal PT may lend credence to the notion that it is better suited for the pulpal treatment of children who cannot return to the dentist for a second appointment due to time constraints or uncooperative behavior. Nevertheless, due to the in vitro nature of this study, additional in vitro and clinical studies are needed to investigate the physical, mechanical, and cytotoxic properties of the materials in order to decide which material may be primarily preferred in clinical applications.

## Figures and Tables

**Table 1:** Type, composition, manufacturer, and lot number of the dental materials utilized in this study.

	Trade Name	Type/Form	Composition	Manufacturer	Lot Number
**Dental** **Restorative Materials**	EverX Flow^TM^	Short fiber-reinforced flowable composite	Bis-MEPP, TEGDMA, UDMA, 140 μm length and 6 μm diameter E-glass fibers, barium glass fiber filler, silicon dioxide	GC, Japan	2104081
	EverX Posterior^TM^	Short fiber-reinforced packable composite	Bis-GMA, TEGDMA, 800 μm length and 17 μm diameter E-glass fibers, barium glass fiber filler, silicon dioxide	GC, Japan	2106171
	Filtek Z550	Nanohybrid composite	Bis-GMA, UDMA, Bis-EMA/PEGDMA/TEGDMA/zirconia and silica fillers	3M ESPE, St. Paul, MN, USA	NC45123
**Calcium** **Silicate Based** **Materials**	NeoMTA 2	Bioceramic MTA/ powder–liquid form	Di- and tricalcium silicate/tantalum oxide/tricalcium aluminate	NuSmile, Houston, TX, USA	2022102806
	NeoPUTTY	Premixed bioceramic MTA/ dough-like form	Di- and tricalcium silicate/calcium aluminate/tantalum oxide/tricalcium aluminate/calcium sulfate/proprietary organic liquid and stabilizers	NuSmile, Houston, TX, USA	2022080402
	TheraCal PT	Resin-modified calcium silicate cement/ flowable form	Base: silicate-glass-mixed cement/polyethylene glycol/dimethacrylate/BisGMA/barium zirconate catalyst: barium zirconate/ytterbium fluoride/initiator	Bisco Inc., Schaumburg, IL, USA	2100000559
**Bonding Agent**	Scotchbond Universal Plus Adhesive Refill	Universal adhesive	10-MDP monomer/dimethacrylate resins/HEMA/methacrylate-modified polyalkenoic acid copolymer/filler/ethanol, water/initiators/silane	3M ESPE Dental Products, St. Paul, MN, USA	8101151

**Table 2:** Evaluation of shear bond strength values between dental restorative materials as a result of immediate and delayed restoration of calcium-silicate-based materials.

		Immediate Restoration	Delayed Restoration	
Calcium-Silicate-Based Materials	Dental Restorative Materials	Shear Bond Strength (MPa) (Mean ± Standard Deviation)	Shear Bond Strength (MPa) (Mean ± Standard Deviation)	
**NeoMTA2**	**EverX Flow^TM^**	4.41 ± 1.73 ^A,a^	10.45 ± 2.35 ^B,b,c^	**t = 17.75** *p* = 0.001 *
	**EverX Posterior^TM^**	4.55 ± 0.66 ^A,a^	7.57 ± 3.20 ^B,b^	**t = 3.47** *p* = 0.007 *
	**Filtek Z550**	3.55 ± 1.55 ^A,a^	14.40 ± 7.12 ^B,c^	**t = 6.15** *p* = 0.001 *
		F = 1.50 P = 0.241	F = 5.29 P = 0.011 *	
**NeoPutty**	**EverX Flow^TM^**	4.71 ± 1.04 ^A,a^	15.04 ± 4.91 ^B,b^	**t = 8.11** *p* = 0.001 *
	**EverX Posterior^TM^**	4.51 ± 1.79 ^A,a^	12.82 ± 4.68 ^B,b,c^	**t = 8.90** *p* = 0.001 *
	**Filtek Z550**	4.37 ± 0.84 ^A,a^	8.94 ± 1.12 ^B,c^	**t = 30.66** *p* = 0.001 *
		F = 1.17 P = 0.843	F = 6.04 P = 0.007 *	
**Theracal PT**	**EverX Flow^TM^**	34.96 ± 6.02 ^A,a,b^	27.34 ± 9.89 ^B,c^	**t = 4.52** *p* = 0.001 *
	**EverX Posterior^TM^**	30.70 ± 6.72 ^A,a^	24.71 ± 7.45 ^B,c^	**t = 4.03** *p* = 0.003 *
	**Filtek Z550**	39.29 ± 7.03 ^A,b^	25.80 ± 5.83 ^B,c^	**t = 12.31** *p* = 0.001 *
		F = 4.23 P = 0.025 *	F = 0.28 P = 0.758	

Notes: Different capital letters represent differences in the row, different lowercase letters represent differences in the column. *p* < 0.05 was accepted as the significance level. * represents *p* values that indicate statistical significance.

**Table 3:** The failure types in the experimental groups.

		Immediate Restoration									Delayed Restoration								
		Dental Restorative Materials									Dental Restorative Materials								
		EverX FlowTM			EverX PosteriorTM			Filtek Z550			EverX FlowTM			EverX PosteriorTM			Filtek Z550		
The Type of Failure		*AF*	*CF*	*MF*	*AF*	*CF*	*MF*	*AF*	*CF*	*MF*	*AF*	*CF*	*MF*	*AF*	*CF*	*MF*	*AF*	*CF*	*MF*
**Calcium** **Silicate** **Based** **Materials**	**NeoMTA2**	-	10	-	-	9	1	-	9	1	6	1	2	6	2	1	-	10	-
	**NeoPutty**	4	6	-	-	8	2	-	10	-	2	6	2	2	6	2	2	6	2
	**Theracal PT**	-	7	3	-	7	3	-	8	2	-	10	-	6	1	3	-	9	1

Abbreviations: AF: adhesive failure; CF: cohesive failure; MF: mixed failure.

## Data Availability

The data presented in this study is available within this article.
